# Development and validation of the coronary heart disease scale under the system of quality of life instruments for chronic diseases QLICD-CHD: combinations of classical test theory and Generalizability theory

**DOI:** 10.1186/1477-7525-12-82

**Published:** 2014-06-04

**Authors:** Chonghua Wan, Hezhan Li, Xuejin Fan, Ruixue Yang, Jiahua Pan, Wenru Chen, Rong Zhao

**Affiliations:** 1 School of Humanities and Management, Guangdong Medical College, Dongguan 523808, China; 2 Shilong Boai Hospital Affiliated to Guangdong Medical College, Dongguan 523325, China; 3 Guangzhou Health Education Institute, Guangzhou 510403, China; 4 The First Affiliated Hospital of Kunming Medical University, Kunming 650031, China; 5 Shenzhen Futian District Institute for Prevention and Control of Chronic Diseases, Shenzhen 518048, China; 6 School of Medicine, Xi’An Jiaotong University, Xi’An 710049, China

**Keywords:** Quality of life, Standardized response mean, Psychometric properties, Intra-class correlations, Multi-trait scaling analysis, Generalizability theory

## Abstract

**Background:**

Quality of life (QOL) for patients with coronary heart disease (CHD) is now concerned worldwide with the specific instruments being seldom and no one developed by the modular approach.

**Objectives:**

This paper is aimed to develop the CHD scale of the system of Quality of Life Instruments for Chronic Diseases (QLICD-CHD) by the modular approach and validate it by both classical test theory and Generalizability Theory.

**Methods:**

The QLICD-CHD was developed based on programmed decision procedures with multiple nominal and focus group discussions, in-depth interview, pre-testing and quantitative statistical procedures. 146 inpatients with CHD were used to provide the data measuring QOL three times before and after treatments. The psychometric properties of the scale were evaluated with respect to validity, reliability and responsiveness employing correlation analysis, factor analyses, multi-trait scaling analysis, t-tests and also G studies and D studies of Genralizability Theory analysis.

**Results:**

Multi-trait scaling analysis, correlation and factor analyses confirmed good construct validity and criterion-related validity when using SF-36 as a criterion. The internal consistency α and test-retest reliability coefficients (Pearson r and Intra-class correlations ICC) for the overall instrument and all domains were higher than 0.70 and 0.80 respectively; The overall and all domains except for social domain had statistically significant changes after treatments with moderate effect size SRM (standardized response mea) ranging from 0.32 to 0.67. G-coefficients and index of dependability (Ф coefficients) confirmed the reliability of the scale further with more exact variance components.

**Conclusions:**

The QLICD-CHD has good validity, reliability, and moderate responsiveness and some highlights, and can be used as the quality of life instrument for patients with CHD. However, in order to obtain better reliability, the numbers of items for social domain should be increased or the items’ quality, not quantity, should be improved.

## Background

Coronary heart disease (CHD) is worldwide the leading cause for morbidity and mortality in adults [1,2]. In Germany, prevalence rates of CHD in the general population are 6.5% (women) to 9.1% (men) [3]. In the United States, CHD is the number 1 cause of death among American men and women, causes 1 of every 5 deaths, and accounted for an estimated $177 billion in direct and indirect costs in 2010 [4]. On the data from National Health and Nutrition Examination Survey 2005 to 2008, an estimated 16300 000 American adults have CHD, with the CHD prevalence for the total, men and women which larger than 20 years old being 7.0%, 8.3% and 6.1%, respectively in the United States [5]. In China, CHD is the second leading cause of cardiovascular death, accounted for 22% of cardiovascular deaths in urban areas and 13% in rural areas [6]. The age-adjusted CHD mortality among the population aged >35 years in 2004 is 128.0 per 100 000 per year for urban men, 97.8 for urban women and 79.7 for rural men, 57.3 for rural women, using the new world standard population [6]. An epidemiological study showed that there were about 1,300,000 new cases of CHD diagnosed in China each year [7], and the incidence of CHD is steadily increasing in China [8]. It was estimated that three-fourths of global deaths and 82% of the total disability adjusted life years lost due to CHD occurred in middle-income countries [9].

There has been a rapid and significant growth in the measurement of quality of life as an indicator of health outcome in patients with CHD, considering that it has long disease duration and much symptoms and therapy side effects. According to WHO (World Health Organization), Quality of Life (QOL) is defined as individual’s perceptions of their position in life in the context of the culture and value systems in which they live and in relation to their goals, expectations, standards and concerns. It includes aspects of health such as physical functioning, social and role functioning, mental health, and general health perceptions that people experience directly. Therefore, QOL is an increasingly important outcome in the study of diseases, and a suitable endpoint in cardiac populations, also in terms of long-term prognosis. In the clinical course of CHD, there are many aspects where patients’ quality of life may be affected which include symptoms of angina and heart failure, limited exercise capacity of the aforementioned symptoms, the physical debility caused, and psychological stress associated with the chronic stress. Many studies have demonstrated that assessing changes in QOL could be a useful complement to clinical management of CHD by assisting in monitoring disease severity and progression [10-12]. Although generic instruments such as the SF-36 and Euroqol EQ-5D were widely used for evaluating QOL of CHD, they do not capture symptoms and side effects specific to CHD [13]. Thus, some disease-specific QOL instruments [13-20] for CHD have been developed including Seattle Angina Questionnaire (SAQ) [14], Quality of Life after Myocardial Infarction (QLMI) [15], the MacNew Heart Disease Quality of Life instrument [16], Minnesota Living with Heart Failure questionnaire (MLHF) [17], Angina Pectoris Quality of Life Questionnaire(APQLQ) [18], the Myocardial Infarction Dimensional Assessment Scale(MIDAS) [19], the Cardiovascular Limitations and Symptoms Profile (CLASP) [20], etc. However, these instruments are appropriate for either angina pectoris or myocardial infarction, and were not developed by the popular modular approach-a general/core module plus specific modules. The modular approach has the advantages of being developed fast and easily, and the resulting scale has well-characterized structure, in which the general module is used to capture the psychometric properties shared by a group of relevant diseases, while the disease-specific module is used to characterize the distinctive disease features [21-23]. Moreover, they are lacking Chinese cultural backgrounds to some extent considering their original use in English-spoken patients. For example, Taoism and traditional medicine focus on good temper and high spirit. Good appetite and sleep are highly regarded in daily life with food culture being very important. This kind of culture dependence does not reflect in most QOL instruments in other languages.

In respond to this need, we have developed a system of Quality of Life Instruments for Chronic Diseases (QLICD, V1.0) by combining a general QOL module and disease-specific modules under the guides of classical test theory (CTT) and Generalizability Theory (GT), Item Response Theory (IRT) [23,24]. The general module, called QLICD-GM, can be used with all types of chronic disease patients, while the specific module addresses the lack of specificity in the general module by capturing the unique aspects of QOL pertaining to the specific disease [23,24]. For example, the Hypertension instrument QLICD-HY is constructed by combining QLICD-GM with the specific module for Hypertension [24]. Similarly, the coronary heart disease instrument (QLICD-CHD) is constructed by combining QLICD-GM with the specific module for this disease. In this paper, we describe the developmental process and study the validation of this QLICD-CHD.

## Methods

### Establishment of the general module (QLICD-GM)

By following WHO’s definition of QOL [25], a nominal group consisting of 16 individuals and a focus group with 10 experts including physicians/nurses and medical researchers were formed to use the programmed decision method to present the conceptual framework and select items [23,24]. The item selection was based not only on qualitative analysis such as nominal group, focus group discussions and in-depth interview, but also on four quantitative statistical procedures—variation procedure, correlation procedure, factor analysis procedure and cluster analysis procedure. The entire process of developing the QLICD-GM has been described in detail elsewhere [23], but the main steps were summarized as a schematic diagram below:

Item pool (73 items)

↓ focus and nominal group discussions

Screened refining Items (46 items)

↓ importance test (86 cases interview), analysis, focus group discussions

Primary scale (V0.0, 38 items)

↓ pre-test (201 cases), analysis, focus group discussions

Final scale (V1.0, 3 domains, 10 facets, 30 items)

↓ 620 patients

Evaluation (validity, reliability, responsiveness)

The final QLICD-GM included 30 items (selected from a 73-item pool) which be classified into 3 domains and 10 facets with physical domain having 8 items (coded PH1-PH8), psychological domain 11 items (coded PS1-PS11) and social domain 8 items (coded SO1-SO8) (see Table 1 for details). This scale was shown to have good validity, reliability, and better responsiveness compared with the SF-36 based on the data from 620 inpatients of seven chronic diseases: hypertension, coronary heart disease, chronic gastritis, peptic ulcer, COPD, chronic obstructive lung disease, and chronic pulmonary heart disease [23].

**Table 1:** Correlation coefficients r among items and domains/facets of QLICD-CHD (n = 146)

**Item**	**Physical domain**			**PHD**	**Psychological domain**				**PSD**	**Social domain**			**SOD**	**Specific domain**			**SPD**
	**IND**	**AAS**	**PHS**		**COG**	**ANX**	**DEP**	**SEC**		**SSS**	**SOE**	**SEF**		**SYM**	**EFM**	**EML**	
PH1	**0.64**	0.34	0.19	**0.52**	0.28	0.23	0.11	0.11	0.21	0.41	0.30	0.04	0.50	0.10	0.11	0.12	0.14
PH3	**0.88**	0.24	0.46	**0.73**	0.51	0.23	0.24	0.31	0.38	0.11	0.47	0.12	0.35	0.37	0.05	0.15	0.29
PH4	**0.85**	0.24	0.52	**0.75**	0.57	0.32	0.30	0.45	0.48	0.05	0.40	0.09	0.27	0.41	0.05	0.21	0.33
PH6	0.28	**0.80**	0.22	**0.50**	0.07	0.01	0.07	0.03	0.02	0.26	0.00	0.00	0.20	0.15	0.23	0.01	0.10
PH7	0.29	**0.81**	0.33	**0.55**	0.24	0.15	0.01	0.09	0.14	0.09	0.11	0.24	0.09	0.18	0.10	0.06	0.12
PH2	0.32	0.30	**0.81**	**0.62**	0.41	0.41	0.46	0.37	0.50	0.06	0.33	0.23	0.20	0.34	0.01	0.23	0.32
PH5	0.30	0.16	**0.80**	**0.56**	0.39	0.28	0.34	0.32	0.40	0.06	0.33	0.08	0.18	0.09	0.13	0.07	0.08
PH8	0.54	0.38	**0.73**	**0.72**	0.46	0.46	0.24	0.29	0.44	0.12	0.33	0.05	0.30	0.59	0.07	0.32	0.49
PS1	0.54	0.18	0.44	0.53	**0.90**	0.34	0.31	0.34	**0.54**	0.04	0.61	0.02	0.34	0.50	0.03	0.32	0.46
PS2	0.48	0.17	0.51	0.52	**0.88**	0.57	0.50	0.50	**0.72**	0.03	0.62	0.13	0.39	0.43	0.17	0.46	0.52
PS5	0.25	0.04	0.33	0.29	0.36	**0.84**	0.64	0.59	**0.75**	0.05	0.37	0.26	0.25	0.19	0.13	0.44	0.38
PS6	0.31	0.14	0.43	0.39	0.48	**0.84**	0.51	0.51	**0.72**	0.03	0.35	0.16	0.23	0.34	0.08	0.48	0.48
PS7	0.27	0.04	0.47	0.35	0.44	**0.87**	0.52	0.53	**0.73**	0.01	0.36	0.24	0.26	0.32	0.08	0.49	0.47
PS3	0.29	0.04	0.39	0.31	0.38	0.54	**0.91**	0.58	**0.74**	0.07	0.35	0.08	0.29	0.22	0.08	0.30	0.31
PS4	0.31	0.03	0.43	0.36	0.47	0.68	**0.90**	0.64	**0.82**	0.09	0.42	0.05	0.34	0.26	0.09	0.37	0.37
PS11	0.13	0.07	0.35	0.19	0.33	0.50	**0.83**	0.67	**0.71**	0.06	0.28	0.22	0.17	0.11	0.08	0.43	0.31
PS8	0.17	0.04	0.23	0.20	0.29	0.35	0.48	**0.79**	**0.58**	0.02	0.22	0.05	0.13	0.33	0.08	0.47	0.45
PS9	0.33	0.03	0.37	0.32	0.32	0.53	0.55	**0.83**	**0.68**	0.05	0.34	0.07	0.27	0.12	0.11	0.29	0.22
PS10	0.37	0.09	0.42	0.41	0.53	0.68	0.74	**0.85**	**0.85**	0.08	0.47	0.25	0.40	0.31	0.18	0.57	0.53
SO2	0.26	0.24	0.07	0.24	0.00	0.07	0.07	0.03	0.06	**0.56**	0.07	0.09	**0.36**	0.01	0.05	0.02	0.01
SO4	0.10	0.10	0.11	0.04	0.07	0.06	0.07	0.05	0.03	**0.77**	0.06	0.16	**0.57**	0.13	0.02	0.08	0.03
SO5	0.11	0.12	0.17	0.02	0.13	0.12	0.05	0.04	0.10	**0.77**	0.11	0.07	**0.53**	0.00	0.02	0.00	0.00
SO7	0.21	0.17	0.10	0.21	0.08	0.12	0.16	0.14	0.15	**0.57**	0.01	0.11	**0.45**	0.18	0.09	0.34	0.31
SO8	0.10	0.09	0.10	0.07	0.29	0.16	0.20	0.14	0.23	**0.61**	0.22	0.07	**0.31**	0.09	0.06	0.03	0.03
SO10	0.34	0.13	0.11	0.27	0.19	0.12	0.22	0.27	0.24	**0.76**	0.18	0.12	**0.71**	0.16	0.01	0.26	0.25
SO1	0.41	0.02	0.33	0.37	0.61	0.37	0.32	0.32	0.48	0.11	**0.82**	0.09	**0.45**	0.33	0.17	0.33	0.39
SO3	0.42	0.02	0.37	0.39	0.49	0.42	0.44	0.51	0.55	0.02	**0.71**	0.26	**0.48**	0.40	0.09	0.49	0.51
SO6	0.41	0.14	0.32	0.39	0.56	0.27	0.24	0.26	0.39	0.04	**0.76**	0.10	**0.47**	0.16	0.18	0.22	0.24
SO9	0.28	0.03	0.26	0.27	0.43	0.25	0.24	0.22	0.33	0.02	**0.74**	0.10	**0.47**	0.07	0.04	0.17	0.14
SO11	0.07	0.15	0.15	0.02	0.06	0.26	0.14	0.15	0.19	0.06	0.18	**1.00**	**0.36**	0.15	0.13	0.39	0.33
CHD1	0.31	0.07	0.52	0.41	0.49	0.35	0.28	0.29	0.42	0.00	0.28	0.21	0.21	**0.62**	0.14	0.40	**0.58**
CHD2	0.21	0.14	0.32	0.29	0.34	0.19	0.23	0.20	0.28	0.10	0.30	0.19	0.30	**0.71**	0.01	0.35	**0.58**
CHD3	0.17	0.13	0.12	0.18	0.35	0.12	0.00	0.08	0.16	0.08	0.17	0.05	0.06	**0.48**	0.04	0.31	**0.43**
CHD4	0.22	0.24	0.18	0.27	0.26	0.17	0.04	0.21	0.20	0.04	0.17	0.02	0.14	**0.78**	0.10	0.33	**0.61**
CHD5	0.32	0.20	0.31	0.36	0.42	0.26	0.17	0.24	0.33	0.12	0.16	0.04	0.20	**0.76**	0.09	0.29	**0.58**
CHD6	0.30	0.11	0.24	0.30	0.30	0.27	0.18	0.26	0.30	0.01	0.15	0.09	0.11	**0.78**	0.13	0.34	**0.63**
CHD7	0.05	0.08	0.09	0.01	0.11	0.11	0.03	0.00	0.08	0.05	0.16	0.13	0.16	0.10	**1.00**	0.22	**0.30**
CHD8	0.00	0.07	0.16	0.05	0.26	0.27	0.16	0.21	0.27	0.03	0.28	0.32	0.21	0.24	0.15	**0.57**	**0.49**
CHD9	0.23	0.19	0.15	0.25	0.16	0.22	0.07	0.18	0.19	0.34	0.03	0.12	0.25	0.22	0.06	**0.31**	**0.29**
CHD10	0.09	0.15	0.27	0.21	0.38	0.42	0.41	0.45	0.50	0.07	0.27	0.26	0.17	0.45	0.11	**0.67**	**0.65**
CHD11	0.02	0.10	0.01	0.05	0.09	0.27	0.21	0.20	0.24	0.05	0.06	0.09	0.02	0.21	0.03	**0.48**	**0.40**
CHD12	0.04	0.10	0.03	0.07	0.11	0.20	0.14	0.15	0.19	0.19	0.08	0.11	0.21	0.04	0.21	**0.48**	**0.34**
CHD13	0.04	0.03	0.21	0.12	0.24	0.42	0.33	0.45	0.44	0.10	0.30	0.35	0.18	0.42	0.15	**0.68**	**0.65**
CHD14	0.00	0.04	0.13	0.04	0.13	0.25	0.20	0.32	0.28	0.04	0.10	0.52	0.20	0.34	0.10	**0.51**	**0.49**
CHD15	0.31	0.00	0.25	0.27	0.57	0.42	0.33	0.39	0.50	0.12	0.55	0.31	0.32	0.34	0.15	**0.56**	**0.53**
CHD16	0.22	0.07	0.00	0.10	0.03	0.08	0.12	0.16	0.12	0.55	0.20	0.02	0.54	0.06	0.13	**0.37**	**0.27**

The numbers in bold are aimed to show strong correlations between items and their own domains/facets easily. PHD: Physical domain, IND: Independence, AAS: Appetite and Sleep, PHS: Physical Symptoms, PSD: Psychological domain, COG: Cognition, ANX: Anxiety, DEP: Depression, SEC: Self-Consciousness, SOD: Social domain, SSS: Social Support/Security, SOE: Social Effects, SEF: Sexual Function, SPD: Specific domain, SYM: Symptom, EFM: Effect of medicine, EML: effect on mental health and daily life.

### Establishment of the specific module

After development of the QLICD-GM, twenty-five items that reflect symptoms, side effects and special mental health of CHD were selected to form the item pool of the specific module. A developmental process similar to the one described above for the general module was used to obtain the final module, which consists of 16 items, coded CHD1-CHD16, classified into 3 facets (see Table 1 in detail). Specifically, the symptom (SYM) facet includes six items of ‘Did you feel short of breath?’ (CHD1), ‘Did you have pain in left shoulder and arm?’ (CHD2), ‘Did you have pain in Upper abdomen?’ (CHD3), ‘Was it last for a long time when your chest pain/discomfort occurs?’ (CHD4), ‘How often has your chest pain/discomfort occur?’ (CHD5), ‘How seriously was it when your chest pain/discomfort occurs?’ (CHD6). The facet of effect of medicine (EFM) includes one item of ‘Can your chest pain been relieved by rest or taking nitroglycerin under the tongue?’ (CHD7). The facet of effect on mental health and daily life (EML) includes nine items of ‘How often have you been worried about chest pain?’ (CHD8), ‘Were you able to control or adjust your negative emotion?’ (CHD9), ‘Did you feel trouble about taking medicine for disease?’ (CHD10), ‘Did you feel trouble about your weight?’ (CHD11), ‘Were you able to adapt to life style change such as low-salt diet and quit smoking?’ (CHD12), ‘Did your disease make you lack of safety?’ (CHD13), ‘Have you been bothered by sexual problem caused by disease?’ (CHD14), ‘How much have the activity limitation cause by your disease affected your life and work?’ (CHD15), ‘If you had to spend the rest of life with the symptoms and treatments the way it is right now, can you accept that optimistically?’ (CHD16).

### Validation of the QLICD-CHD

#### Data collection and scoring

The formal QLICD-CHD (the general module QLICD-GM plus the specific module) described above was used for patients with CHD in a field survey in order to study its psychometric properties (validity, reliability and responsiveness). The study population was limited to CHD inpatients who were able to read and understand the questionnaires at any stages and treatments. The participating investigators (doctors and medical post-graduate students) explained the trial and the scale to the patients and obtained informed consent from those who agreed to participate in the study. Each patient (n = 146) was asked to answer the questionnaires at the time of admission to the hospital by themselves. A random sub-sample consisting of 50 patients also participated in a second assessment the following day after hospitalization so that the test-retest reliability can be calculated. All patients available at the third scheduled assessment time-point (111 cases) completed the measures at discharge (after approximately 1 week of treatment) to evaluate responsiveness of the questionnaire. Answers were checked immediately each time by the investigators in order to ensure its integrality. If missing values were found, the questionnaire would be returned to the patients to fill in the missing item.

The Chinese version of SF-36 [26], which have eight domains: Physical Function (PF), Role-Physical (RP), Bodily Pain (BP), General Health (GH),Vitality (VT), Social Function (SF), Role-Emotional (RE) and Mental-Health (MH), was also used to provide data for assessing the criterion-related validity of the QLICD-CHD, and also convergent and discriminant validity.

Based on the data collected, the raw scores of items, domains and overall scale were calculated. Each item of QLICD-CHD is rated in a five-level Likert scoring system, namely, not at all, a little bit, somewhat, quite a bit, and very much. The positively stated items are directly scored from 1 to 5, while the negatively stated items are reversely scored. Each domain score is obtained by adding together the within-domain item scores. The overall scale score is the sum of the four domain scores.

For comparison purposes, all domain scores were linearly converted to a 0–100 scale using the formula: SS = (RS-Min) × 100/R, where SS, RS, Min and R represent the standardized score, raw score, minimum score, and range of scores, respectively.

### Statistical analysis for psychometrics

The validity, reliability, and responsiveness of the QLICD-CHD were analyzed. Validity is the degree to which the instrument measures what it is supposed to measure, with several types of validity being distinguished [27,28]. Construct validity was evaluated by Pearson’s correlation coefficient r (item-domains/facets correlations) as well as by factor analysis with Varimax Rotation. Multi-trait scaling analysis [29] was employed to test item convergent and disciminant validity, with the two criteria: (1) convergent validity is supported when an item-domain correlation is 0.40 or greater; (2) disciminant validity is revealed when item-domain correlation is higher than that with other domains. Criterion-related validity was evaluated by correlating corresponding domains of the QLICD-CHD and SF-36 because of the lack of an agreed-upon gold standard. Relatively high correlations among conceptually related domains and relatively low correlation among conceptually distinct domains would suggest high criterion-related validity. And this can also demonstrate convergent and discriminant validity because they involve comparing logically related measures to see if they are correlated more strongly (convergent) or more weakly (discriminant).

Reliability is the degree to which an instrument is free from random error, with being evaluated by measuring internal consistency reliability and reproducibility frequently. The internal consistency, which refers to the homogeneity of the items of the scale, was assessed by Cronbach’s alpha coefficient for each domain/facet. A high internal consistency suggests that the scale is measuring a single construct. Reproducibility (the test-retest reliability) establishes the stability of an instrument over time in a stable population [28]. It was evaluated by the Pearson’s correlation coefficients between the first and second assessments, and intra-class correlation (ICC) with definition of absolute for single measure under the two-way mixed model [30,31]. Patients were considered stable if they did not experience treatments the following day to hospital.

Responsiveness is the instrument’s ability to detect clinically important change over time. It was measured by comparing the mean score change between the two assessments before and after treatments using paired *t*-tests as well as the effect size, SRM (standardized response mean) [32,33].

### Generalizability theory analysis

Besides classical test theory analysis above, we also applied Generalizability Theory (G theory) to investigate the score dependability of the QLICD-CHD. G theory has been presented as a way to refine the designs of measurement procedures in an attempt to yield reliable data [34-37]. Serving as an alternative to the more familiar classical measurement theory, which yields the less useful intra-class correlation coefficients, G theory addresses the dependability of measurements and allows for the simultaneous estimation of multiple sources of variance, including interactions. Thus, a distinction is made between 2 types of studies: G studies and D studies. A G study quantifies the amount of variance associated with the different facets (factors) that are being examined. A D study provides information about which protocols are optimal for a particular measurement situation by generating Generalizability (G) coefficients that can be interpreted as reliability coefficients across various facets of the study.

In our research, G-Studies and D-Studies were performed to estimate the variance components and dependability coefficients in one facet person-by-item design (p × i design). We defined the quality of life of patients as the target of measurement and items as one facet of measurement error. Given every person is asked to reply to all items, the design is One-facet Crossed Design [34-37]. For the G-Study, a universe of admissible observations, which consists of the object of measurement and the measurement error facets, is defined and the variance components are estimated. For the D-Study, a universe of admissible generalizations, which represents the measurement conditions based on the object of measurement and the measurement facets a researcher is willing to generalize over, is defined and the variance components associated with the universe of admissible generalizations are estimated.

## Results

### Socio-demographic and clinical characteristics of the sample

The 146 patients with CHD varied in age from 19 to 80, with median age of 64.0 and mean age 62.1 ± 11.3. Among the study patients, 132 (90.4%) were of Han ethnicity and others were of minority including Yi, Bai, Hui, etc., the majority were married (128 cases, 87.7%) while 18 (12.3%) were single and others. On gender and education level, 105 cases (71.9%) were male while 41 (28.1%) were female, 37 (25.3%) finished primary school, while 75 (52.4%) completed high school, and 32 (22.0%) had a college or post-graduate degree. Distribution of occupations was as follow: workers 20.5% (30 cases), farmer 9.6% (14), teacher 7.5% (11), cadre 19.9% (29), and others 42.5% (62). With regard to perceived income, 20 (13.7%) were in poor, 93 (63.7%) in fair, and 33 (22.6%) in high. Regarding medical insurance, self-paid accounted in 16 cases (11.0%) and partly public insurance/ public insurance accounted in 130 cases (89.0%). In terms of clinical types, angina pectoris were 97 cases (67.4%) while myocardial infarctions were 49 cases (32.6%).

### Construct validity

Correlation analyses showed that there were strong associations between items and their own domains/facets (most correlation coefficients are higher than 0.5), but weak relationship between items and other domains/facets (see Table 1). For example, correlation coefficients between PHD and items of PH1-PH8 (in bold) are higher than those between PHD and other items. Especially, the correlation coefficients between items and their own facets were much larger than that between items and other facets.

There were 8 principal components (initial eigenvalues >1) abstracted from 30 items of the general module (QLICD-GM) by factor analysis, accounting for 68.1% of the cumulative variance. By using the Varimax rotation method, it can be seen that the 8 principal components reflected different facets under three domains of the general module. Specifically, the fourth and fifth principal components mainly represented the physical domain with higher loadings on PH5(0.69), PH7(0.64) and PH8(0.72); The second and third principal components largely reflected the social domain with higher loadings on SO1(0.76), SO4(0.78), SO5(0.83), SO6(0.76), SO8(0.62) and SO10(0.83); The other principal components generally depicted the psychological domain with higher loadings on PS3(0.77), PS4(0.81), PS5(0.71), PS8(0.65), PS9(0.69), PS10(0.80) and PS11(0.79).

Similarly, the principal component factor analysis extracted 4 principal components from the 16 items of the specific module with the cumulative variance of 60.6%, reflecting 3 facets of this module. And here the first principal component represented the facet of treatment side-effects with higher factor loadings on CHD2(0.60),CHD3(0.64), CHD4(0.83), CHD5(0.83) and CHD6(0.82), the second and the fourth principal components captured the facet of mental and physical health with higher factor loadings on CHD8(0.68), CHD9(0.65), CHD12(0.72), CHD13(0.66), CHD15(0.70) and CHD16(0.66).

From results above, theoretical construct was confirmed generally by data analysis, showing good construct validity.

### Criterion-related validity

Correlation coefficients among the domain scores of the QLICD-CHD and SF-36 were presented in Table 2, showing that the correlations between the same and similar domains are generally higher than those between different and non-similar domains. For example, the coefficient between the physical domain of QLICD-CHD and physical function of SF-36 was 0.61, higher than any other coefficients in this row. Similarly, the coefficient between the psychological domain of QLICD-CHD and mental health of SF-36 was 0.49, higher than any other coefficients in this row (PCS and MCS are not mutually exclusive domain of SF-36).

**Table 2:** Correlation coefficients among domains scores of QLICD-CHD and SF-36 (n = 146)

**QLICD-CHD**	**SF-36**							
	**PF**	**RP**	**BP**	**GH**	**VT**	**SF**	**RE**	**MH**
**PHD**	0.61	0.30	0.36	0.39	0.53	0.37	0.33	0.32
**PSD**	0.46	0.28	0.36	0.45	0.44	0.42	0.34	0.49
**SOD**	0.37	0.30	0.29	0.24	0.46	0.28	0.28	0.40
**SPD**	0.32	0.19	0.28	0.35	0.26	0.24	0.28	0.35

PHD: physical domain, PSD: psychological domain, SOD: social domain, SPD: specific domain.

PF: physical function, RP: role-physical, BP: bodily pain, GH: general health, VT: vitality, SF: social function, RE: role-emotional, MH: mental-health.

These confirmed the criterion-related validity to a reasonable degree and also demonstrated the convergent and divergent validity to some extent.

### Reliability

The reliability of the scale was evaluated by three procedures: internal consistency, test-retest and ICC (see Table 3 for details). The Cronbach's α for all domains and facets were computed using the measurements data at admission because of larger sample size. As can be seen in Table 3, the Cronbach's α for these four domains were higher than 0.70, and most of them were higher than 0.70 at the facet levels.

**Table 3:** **Reliability of the quality of life instrument QLICD-CHD (n = 146 for** α**, n = 50 for *r *and ICC)**

**Domains/Facets**	**Internal consistency coefficient α**	**Test-retest reliability correlation coefficient *r***	**Test-retest reliability ICC (95% CI )**
**Physical domain (PHD)**	0.76	0.95	0.95 (0.91-0.97)
Independence	0.70	0.98	0.98 (0.97-0.99)
Appetite and sleep	0.46	0.77	0.77 (0.62-0.86)
Physical Symptoms	0.67	0.97	0.98 (0.96-0.99)
**Psychological domain (PSD)**	0.90	0.92	0.92 (0.86-0.95)
Cognition	0.73	0.92	0.92 (0.87-0.96)
Anxiety	0.81	0.85	0.84 (0.74-0.91)
Depression	0.84	0.93	0.93 (0.88-0.96)
Self-consciousness	0.76	0.91	0.91 (0.84-0.95)
**Social domain (SOD)**	0.63	0.86	0.85 (0.75-0.91)
Social Support/Security	0.74	0.84	0.83 (0.71-0.90)
Social Effects	0.75	0.92	0.91 (0.85-0.95)
Sexual function	-	0.94	0.96 (0.94-0.98)
**Sub-total (QLICD-GM)**	-	0.92	0.92 (0.86-0.95)
**Specific domain (SPD)**	0.79	0.80	0.80 (0.68-0.88)
Symptom	0.78	0.70	0.72 (0.54-0.73)
Effect of medicine	-	0.61	0.61 (0.40-0.76)
Effect on mental health and daily life	0.65	0.89	0.92 (0.86-0.95)
**Total (TOT)**	-	0.90	0.91 (0.84-0.95)

- not acceptable/suitable, ICC: intra-class correlation, CI: confidence interval.

The test-retest correlation coefficients (r) for the 4 domains and 13 facets of QLICD-CHD ranged between 0.61-0.98, with r = 0.90 for the overall scale and the minimum r = 0.80 for SPD among the four exclusive domains. The differences in domain and facet scores between the first and the second assessments were not statistically significant for all domains and most facets except for Independence and symptom by paired t tests (*P* > 0.05). The results from ICC were very similar to Pearson’s correlation coefficients (r).

### Reliability from generalizability theory

The estimated G-study results were provided in Table 4 based on the current design, in which 146 patients filled out the quality of life instrument QLICD-CHD with 46 items. For physical domain, the variances accounted for 67.22% by person-by-item interactions and 28.30% by person, only a small source of variation (4.48%) was due to item. Given the largest source of variation in this domain score is by the person-by-item interaction, it means that different people might understand and react to the same item in different ways despite having the same total score on the scale. Similarly, the largest source of variation was due to person-by-item interactions in other domains, while the variances by person were in the second place (except for social domain by item).

**Table 4:** The estimated variance components and percentage of variance for p × i design in G-study for four domains of quality of life instrument QLICP-CHD

	***p*** **(person)**		***i*** **(item)**		p * i (person*item)	
**Domain**	**Variance component**	**Percent (%)**	**Variance component**	**Percent (%)**	**Variance component**	**Percent (%)**
PHD	0.50	28.30	0.08	4.48	1.20	67.22
PSD	0.59	42.20	0.15	10.87	0.65	46.93
SOD	0.21	11.76	0.25	14.33	1.30	73.91
SPD	0.26	17.45	0.16	10.74	1.07	71.81

p: person effect, i: item effect, p × i: person-by-item interaction effect.

The D-Studies were performed to estimate G-coefficients and Ф coefficients for the current design and alternative designs with varied numbers of items for four domains of QLICP-CHD, with results presenting in Table 5. It showed acceptable reliability coefficients (G and Ф coefficients >0.70) for three of four domains except for social domain for the current design. In addition, Table 5 showed the effects of the various levels of items (from 6 to 22) on reliability with G ranging from 0.59 to 0.92, and Ф ranging from 0.55 to 0.91.

**Table 5:** G-coefficients and Ф-coefficients for different numbers of items for p × I design in D-study for four domains of quality of life instrument QLICP-CHD

**Domain**	**Number of items**	**σ**^**2**^**(P)**	**σ**^**2**^**(I)**	**σ**^**2**^**(PI)**	**σ**^**2**^**(δ)**	**σ**^**2**^**(∆)**	**σ**^**2**^**(×**_**PI**_**)**	**σ**^**2**^**(Eρ**^**2**^**)**	**Φ**
Physical domain	6	0.51	0.01	0.20	0.20	0.21	0.02	0.72	0.70
	**8**	**0.51**	**0.01**	**0.15**	**0.15**	**0.16**	**0.02**	**0.77**	**0.76**
	10	0.51	0.01	0.12	0.12	0.13	0.01	0.81	0.80
	12	0.51	0.01	0.10	0.10	0.11	0.01	0.84	0.83
	14	0.51	0.01	0.09	0.09	0.09	0.01	0.86	0.85
Psychological domain	9	0.59	0.02	0.07	0.07	0.09	0.02	0.89	0.87
	10	0.59	0.02	0.07	0.07	0.08	0.02	0.90	0.88
	**11**	**0.59**	**0.01**	**0.06**	**0.06**	**0.07**	**0.02**	**0.91**	**0.89**
	12	0.59	0.01	0.06	0.06	0.07	0.02	0.92	0.90
	13	0.59	0.01	0.05	0.05	0.06	0.02	0.92	0.91
Social domain	9	0.21	0.03	0.14	0.14	0.17	0.03	0.59	0.55
	**11**	**0.21**	**0.02**	**0.12**	**0.12**	**0.14**	**0.03**	**0.64**	**0.60**
	13	0.21	0.02	0.10	0.10	0.12	0.02	0.67	0.63
	15	0.21	0.02	0.09	0.09	0.10	0.02	0.71	0.67
	17	0.21	0.02	0.08	0.08	0.09	0.02	0.73	0.70
Specific domain	14	0.26	0.01	0.08	0.08	0.09	0.01	0.77	0.75
	**16**	**0.26**	**0.01**	**0.07**	**0.07**	**0.08**	**0.01**	**0.80**	**0.77**
	18	0.26	0.01	0.06	0.06	0.07	0.01	0.81	0.79
	20	0.26	0.01	0.05	0.05	0.06	0.01	0.83	0.81
	22	0.26	0.01	0.05	0.05	0.06	0.01	0.84	0.82

σ^2^(δ) is the variance components of relative error.

σ^2^(∆) is the variance components of absolute error.

σ^2^(×_PI_) is the variance components of error when estimating the universe score by using sample mean.

σ^2^(Eρ^2^) is the Generalizability coefficient.

Φ is the index of dependability.

### Responsiveness

It can be seen in Table 6 that significant changes occurred for domains of physical, psychological and the specific, and also the sub-total (QLICD-GM) and overall scale (P < 0.01) with effect size SRM ranging from 0.32 to 0.67. At the facets level, five of thirteen facets were of statistical significance with effect size SRM ranging from 0.20 to 0.88.

**Table 6:** **Responsiveness of the quality of life instrument QLICD-CHD (**$\bar{x}\pm s$**) (n = 111)**

**Domains/Facets**	**Before treatment**	**After treatment**	**Differences**	**SRM**	***t***	***p***
**Physical domain**	53.60 ± 20.37	58.87 ± 17.72	-5.26 ± 12.46	0.42	-4.45	0.000
Independence	60.51 ± 28.49	64.49 ± 26.03	-3.98 ± 18.42	0.22	-2.28	0.025
Appetite and Sleep	46.62 ± 25.61	50.45 ± 23.83	-3.83 ± 23.04	0.17	-1.75	0.083
Physical Symptoms	51.35 ± 23.69	58.86 ± 19.92	-7.51 ± 14.82	0.51	-5.34	0.000
**Psychological domain**	70.52 ± 20.51	75.63 ± 17.78	-5.12 ± 16.20	0.32	-3.33	0.001
Cognition	57.55 ± 28.96	63.74 ± 28.50	-6.19 ± 19.79	0.31	-3.30	0.001
Anxiety	63.81 ± 25.16	72.22 ± 20.11	-8.41 ± 20.21	0.42	-4.38	0.000
Depression	78.23 ± 23.52	82.21 ± 18.95	-3.98 ± 19.78	0.20	-2.12	0.036
Self-Consciousness	78.15 ± 22.22	80.41 ± 20.43	-2.25 ± 18.50	0.12	-1.28	0.202
**Social domain**	62.29 ± 14.48	62.18 ± 14.50	0.10 ± 8.36	0.01	0.13	0.898
Social Support/Security	68.73 ± 20.20	69.71 ± 19.51	-0.98 ± 13.98	0.07	-0.74	0.464
Social Effects	53.83 ± 24.24	52.48 ± 24.41	1.35 ± 16.63	0.08	0.86	0.394
Sexual Function	57.43 ± 31.00	55.86 ± 32.33	1.58 ± 21.13	0.07	0.79	0.433
**Sub-total (QLICD-GM)**	62.99 ± 14.56	66.23 ± 13.50	-3.24 ± 9.61	0.34	-3.55	0.001
**Specific domain**	61.51 ± 14.00	70.12 ± 13.32	-8.60 ± 12.91	0.67	-7.02	0.000
Symptom	64.90 ± 18.96	84.27 ± 19.27	-19.37 ± 22.00	0.88	-9.27	0.000
Effect of medicine	47.30 ± 23.92	60.59 ± 31.18	-13.29 ± 32.83	0.40	-4.27	0.000
Effect on mental health and daily life	60.84 ± 15.38	61.74 ± 14.40	-0.90 ± 11.95	0.08	-0.79	0.429
**Total (TOT)**	62.48 ± 13.22	67.58 ± 12.27	-5.11 ± 9.50	0.54	-5.67	0.000

## Discussions

### On development approach and advantages

Since same-class diseases such as cancers share many things in common, an approach widely adopted in recent years to develop QOL instruments for diseases within a common class is to combine a general module for the entire class of disease with specific modules for individual diseases to capture both common features within the disease class and disparities among different disease members. This approach can substantially reduce the amount of time and effort in developing new instruments. Both the QLQs from EORTC and the FACTs from CORE for QOL assessments of cancer patients have been developed based on this modular principle [21,38]. Unlike these two QOL instruments systems, we employed this modular approach to systematically and more efficiently develop a system of new instruments for chronic diseases directly, with QLICD-GM forming the general module and QLCID-CHD being a specific scale for coronary heart disease. This modular approach unifies all disease-specific instruments of QLICDs using the same general module with similar constructs. To our knowledge, although a number of instruments have been widely used for studying CHD impacts on patients’ QOL, no one was developed directly by the modular approach. Therefore, the QLICD-CHD has several advantages over existing instruments [23,24]. First, it can compare HRQOL across diseases by the general module and also capture the symptoms and side effects by the specific module, demonstrating both generic and specific properties. Second, it consists of a moderate number of items with a clear hierarchical structure (items → facets → domains → overall) so that mean scores can be computed not only at the domain (four domains) and the overall levels but also at the different facet levels (13 facets) to detect changes in greater detail. Users can select either one or both levels for a study at hand. Third and perhaps more important is the strong Chinese cultural background underlying the QLICD-CHD. For example, the Chinese culture pays more attention to family relationship and kinship, dietary, temperament and high spirit, which are probed by the items of QLICD-CHD focusing on this type of cultural heritage such as appetite, sleep, energy and family support. Specifically, items of PH6 ‘Have you had a good appetite?’, PH7 ‘Were you satisfied with your sleep?’, PH2 ‘Have you felt fatigue easily?’, SO4 ‘Have you had good relations with your families?’, and SO5 ‘Could you acquire material and emotional help and support from your family when you need?’ etc. reflected these aspects in details.

### On psychometrics

Generally, a practical QOL instrument must be validated with respect to at least three aspects: validity, reliability and responsiveness. Instrument validity is the extent to which an instrument can capture what it purports to measure. By following WHO’s definition of QOL and the programmed decision procedures, we developed the QLICD-CHD by using focus group discussion, in-depth interview and pre-testing to effectively reduce the number of items in the final version to 30 from an initial 73 item pool for the general module, and to 16 from an initial pool of 25 items for the specific module, ensuring good content validity and sound conceptual structure. Correlation analyses showed strong association between items and their own domains/facets but weak correlations between items and other domains/facets. Factor analysis revealed that the components extracted from the data basically coincide with the theoretical construct of the instrument. These results confirmed the good construct validity. Correlation coefficients between domain scores of QLICD-CHD and SF-36 showed the criterion-related validity to a reasonable degree and the convergent and divergent validity to some extent.

Reliability refers to the reproducibility or consistency of item scores from one assessment to another. Test-retest reliability (r), ICC and internal consistency (Cronbach’s α) are the most frequently used indicators and were tested in the current study. It is well recognized that internal consistency (α) should be at least 0.70 and reliability (r) should be above 0.80 in a test–retest situation [32]. Thus, our results in Table 3 showed that this instrument has good reliability for all α were higher than 0.70 and r (ICC) greater than 0.80 at domain levels.

The assessment methods on responsiveness can be generally divided into two categories: internal and external [32,33]. In this paper we focused on internal responsiveness with the hypothesis that the sensitive instrument should detect changes when they occur after treatment. SRM is a good indicator of effect size, with values of 0.20, 0.50 and 0.80 representing small, moderate and large responsiveness [32,33]. As seen from Table 6, QOL scores had significant changes after treatment for three of the four domains as well as the overall score (P < 0.05), with SRM equal to 0.52, 0.28, 0.62 and 0.56. Given that it reasonable to expect no statistically significant change for the social domain and some facets pertaining to stable traits post-treatment, QLICD-CHD seems to have good responsiveness.

### On analysis of generalizability theory

Traditionally, the scale is assessed by classical test theory analysis, in this research Generalizability Theory was also applied both in G-study and D-study. Which coefficients will be selected depending on the researchers’ interests? If one’s interest lies in ranking people (relative decision), then the G-coefficient informs about how dependable a score is. If one’s interest lies in the absolute standings to a criterion (absolute decision), the index of dependability Ф reflects the score dependability. The index of dependability is typically lower than G-coefficients because they consider the main error effects in addition to the interaction effects that are used for G-coefficients. This research presented both G-coefficients and Ф, and also their changes when items assumed to be changed. For social domain, we estimated a G-coefficient of 0.64 and an index of dependability of 0.60 for the current design, which was a little below the acceptable level of 0.70. Hence, the domain’s items need improvement. For an alternative design with 17 items, the G-coefficient estimated to be 0.74 and the index of dependability 0.70. Therefore, it will be better to increase the numbers of items of social domain from 11 to 17 in order to reach an acceptable dependability. For other domains, G- coefficients and index of dependability were all greater than 0.70 for the current design, and changed a little as items changing. It can be considered that current items are reasonable and acceptable for these domains.

To sum up, the analysis from Generalizability Theory confirmed the reliability of the scale further. However, the numbers of items for social domain should be increased in order to obtain better reliability.

### Study limitations

It is worthy to note that the sample size of the study is not very large, which may also affect the findings, especially those with respect to factor analysis (146 cases vs 30 variables for the general module). Although correlational analysis was conducted simultaneously to display the construct, which overcome it to some extent, additional larger studies are needed to validate it further. Moreover, the subjects in this study were selected from the inpatient population at hospitals. Additional studies are needed to assess the generalizability of the instrument to other settings and populations such as outpatients at a local clinic.

In summary, the QLICD-CHD can be used as a useful instrument in measuring and assessing quality of life for patients with coronary heart disease who speak Chinese (the largest population in the world), with good psychological properties and some highlights.

## Competing interests

The authors declare that they have no competing interests.

## Authors’ contributions

WC, HL and CW designed the study. RY, XF, RZ, JP performed the data collection and RY, RZ and CW performed data analyses, and all authors contributed to interpreting the data. WC and CW wrote the first draft, which was critically revised by all others. All authors have read and approved the final manuscript.

## Authors’ information

Chonghua Wan and Hezhan Li are as the first co-author with the same contributions.
